# Density, abundance, survival, and ranging patterns of common bottlenose dolphins (*Tursiops truncatus*) in Mississippi Sound following the *Deepwater Horizon* oil spill

**DOI:** 10.1371/journal.pone.0186265

**Published:** 2017-10-20

**Authors:** Keith D. Mullin, Trent McDonald, Randall S. Wells, Brian C. Balmer, Todd Speakman, Carrie Sinclair, Eric S. Zolman, Fawn Hornsby, Shauna M. McBride, Krystan A. Wilkinson, Lori H. Schwacke

**Affiliations:** 1 Southeast Fisheries Science Center, National Marine Fisheries Service, National Oceanic and Atmospheric Administration, Pascagoula, Mississippi, United States of America; 2 Western Ecosystems Technology, Inc., Cheyenne, Wyoming, United States of America; 3 Sarasota Dolphin Research Program, Chicago Zoological Society, % Mote Marine Laboratory, Sarasota, Florida, United States of America; 4 Hollings Marine Laboratory, National Centers for Coastal Ocean Science, National Ocean Service, National Oceanic and Atmospheric Administration, Charleston, South Carolina, United States of America; University of Missouri Columbia, UNITED STATES

## Abstract

After the *Deepwater Horizon* (DWH) oil spill began in April 2010, studies were initiated on northern Gulf of Mexico common bottlenose dolphins (*Tursiops truncatus*) in Mississippi Sound (MSS) to determine density, abundance, and survival, during and after the oil spill, and to compare these results to previous research in this region. Seasonal boat-based photo-identification surveys (2010–2012) were conducted in a section of MSS to estimate dolphin density and survival, and satellite-linked telemetry (2013) was used to determine ranging patterns. Telemetry suggested two different ranging patterns in MSS: (1) inshore waters with seasonal movements into mid-MSS, and (2) around the barrier islands exclusively. Based upon these data, dolphin density was estimated in two strata (Inshore and Island) using a spatially-explicit robust-design capture-recapture model. Inshore and Island density varied between 0.77–1.61 dolphins km^−2^ ($\bar{x}$ = 1.42, 95% CI: 1.28–1.53) and 3.32–5.74 dolphins km^−2^ ($\bar{x}$ = 4.43, 95% CI: 2.70–5.63), respectively. The estimated annual survival rate for dolphins with distinctive fins was very low in the year following the spill, 0.73 (95% CI: 0.67–0.78), and consistent with the occurrence of a large scale cetacean unusual mortality event that was in part attributed to the DWH oil spill. Fluctuations in density were not as large or seasonally consistent as previously reported. Total abundance for MSS extrapolated from density results ranged from 4,610 in July 2011 to 3,046 in January 2012 ($\bar{x}$ = 3,469, 95% CI: 3,113–3,725).

## Introduction

The *Deepwater Horizon* (DWH) oil spill in the northern Gulf of Mexico (GoM) began on 20 April 2010 and over the next 87 days, 3.19 million barrels of oil (~500,000 m^3^) flowed into the GoM. It was the largest marine oil spill in United States history and at its maximum extent covered an area of 40,000 km^2^ which impacted vast swathes of oceanic, coastal, and estuarine waters from the Texas-Louisiana border to the central Florida Panhandle [1–4]. Studies were initiated to assess the impacts of the DWH oil spill on a wide range of plant and animal species, including cetaceans, as part of a Natural Resources Damage Assessment (NRDA) under the Oil Pollution Act of 1990 [3, 5]. Cetaceans in the northern GoM inhabit oceanic (>200 m deep; 20 species), outer continental shelf (20–200 m deep; 2–3 species), and coastal (0–20 m deep; 1 species) waters as well as bays, sounds, and estuaries (BSEs; 1 species) [6]. In many of these habitats, including BSE waters, cetaceans encountered DWH oil and potentially inhaled, directly aspirated, or ingested the oil or its toxic components [5, 7].

NRDA field studies in oceanic, outer continental shelf, and coastal waters—aimed at understanding potential exposure of cetaceans by estimating abundance and distribution patterns of cetaceans in relation to the DWH oil—consisted primarily of aerial- and ship-based surveys and satellite-linked telemetry [5]. The common bottlenose dolphin (*Tursiops truncatus*) is the only cetacean species that inhabits northern GoM BSEs [8] where a wider range of field techniques were employed to assess impacts associated with the DWH oil spill. These field techniques included: photo-identification (photo-ID) capture-recapture surveys to estimate abundance and survival; satellite-linked telemetry to study ranging and movements patterns; capture-release health assessments; remote biopsy sampling to study stock structure, contaminant loads, stable isotopes and reproductive hormones; and targeted surveys to relocate specific individuals for monitoring of reproductive outcomes (e.g. [9–12]).

**Fig 1:**
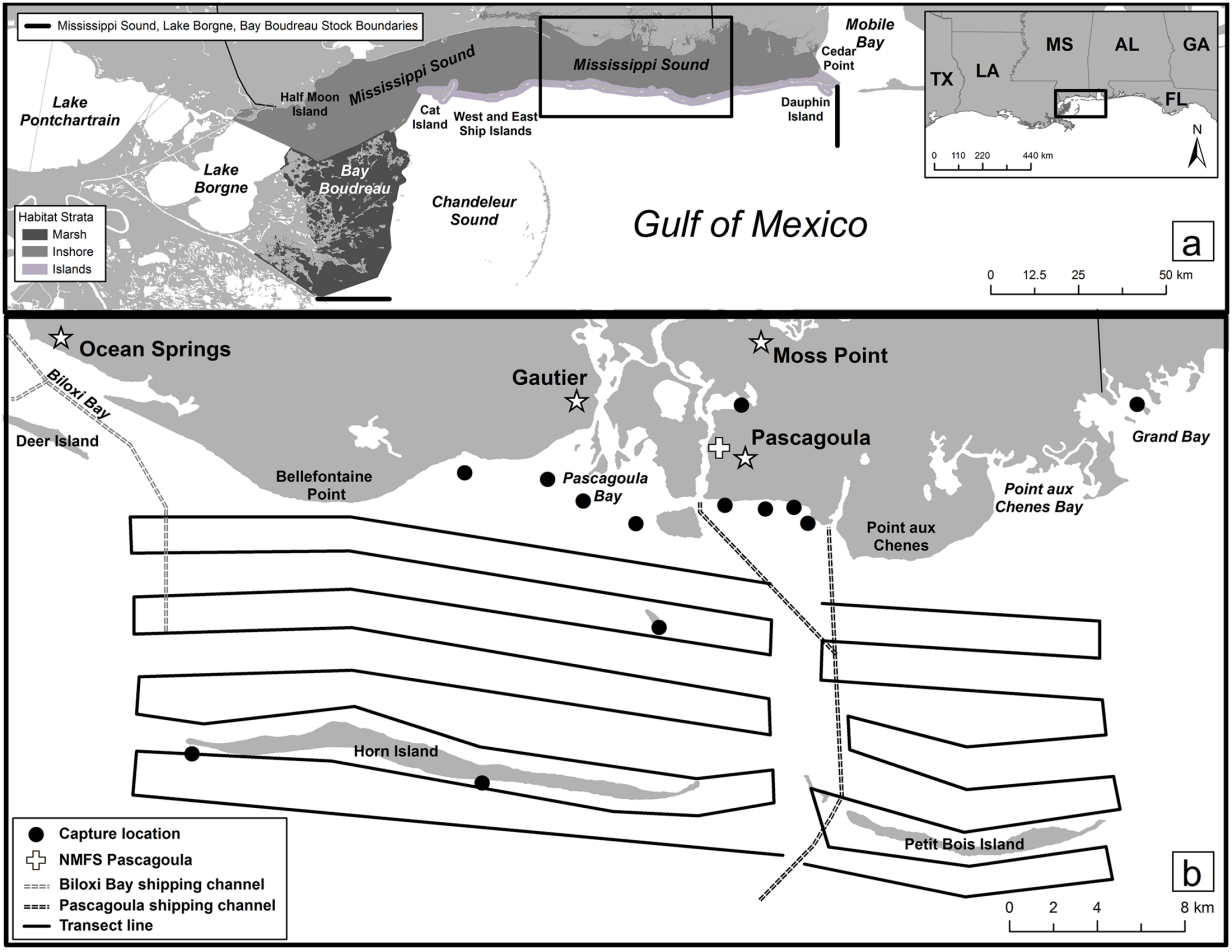
Map of the Mississippi Sound region and common bottlenose dolphin study area in the northern Gulf of Mexico. a. The Mississippi Sound (2,129 km^2^), Lake Borgne (730 km^2^; area west of Half Moon Island), Bay Boudreau (778 km^2^; Marsh) common bottlenose dolphin stock area, surrounding waters, and the habitat strata (Inshore and Island) for estimating density and abundance (Island stratum − waters <1 km from a barrier island and a 2-km wide corridor in the passes between islands; Inshore stratum—waters north of the Island stratum and Bay Boudreau). b. Mississippi Sound study area with east-west transect lines for capture-recapture photo-identification surveys, and capture locations of 19 satellite-linked tagged dolphins (capture locations may represent multiple tagged dolphins).

The U.S. National Marine Fisheries Service (NMFS) has defined 31 BSE common bottlenose dolphin stocks in the northern GoM from the Texas-Mexico border to the Florida Keys, where a stock is assumed to be a demographically independent unit [13, 14]. In an attempt to cover the geographic range of BSE stocks potentially impacted by the DWH oil spill, in June 2010, studies were initiated in Mississippi Sound, MS (MSS), St. Joseph Bay, FL, and as defined by Michel et al. [2], heavily oiled Barataria Bay, LA. Barataria Bay and St. Joseph Bay are each defined as one stock and MSS is the primary component of the area defined for one stock that also includes Lake Borgne and Bay Boudreau (Fig 1) [13]. Studies in St. Joseph Bay were discontinued after February 2011 due to minimal oiling in that area, but studies in MSS and Barataria Bay, continued through 2014 and 2015, respectively. Here we report on dolphin abundance, survival, and ranging patterns based on NRDA studies in MSS. Results of similar studies from Barataria Bay have been reported elsewhere [12, 15].

Since the mid-1970s, the MSS dolphin population has been one of the most consistently studied marine mammal populations in the northern GoM (e.g. [16–20]). The impetus for studies conducted in MSS before 1990 was management concerns related to the live-capture industry that supplied common bottlenose dolphins for public display, research, and military applications. MSS was the site of the largest live-capture fishery for dolphins in North America [21] but no dolphins have been removed since 1989 [22]. In general, MSS dolphin abundance, estimated from line-transect surveys, appears to fluctuate seasonally with about twice as many dolphins in the warmer season as during the colder season [13, 18, 19, 23]. Similar to other northern GoM BSEs, there is evidence of site fidelity across seasons and years as well as both year-round and seasonal dolphin residence in MSS, based on photo-ID studies and periodic resightings of animals freeze-branded in the early 1980s [19, 24–27]. However, because of the large number of dolphins and its extensive geographic scope, definitive conclusions on residence patterns and site fidelity have been difficult to make for MSS compared to other smaller GoM BSEs.

Multiple capture-release health assessment studies were conducted in Barataria Bay (August 2011, June 2013, and June 2014), and reported a high prevalence of pulmonary disease, impaired stress response, and poor body condition, all of which have been related to oil or petroleum-associated compounds in experimental studies of other mammal species (see [11] for discussion). Although many of the observed health issues in Barataria Bay dolphins improved in the 2013 and 2014 health assessment studies, some conditions, such as the pulmonary disease and abnormal stress response, were still seen [28]. A single capture-release study was conducted in MSS in July/August 2013, and reported results similar to those from Barataria Bay dolphins the same year. Specifically, MSS dolphins in 2013 were also found to have a high prevalence of moderate-severe lung disease, and abnormally low serum cortisol levels indicative of an impaired stress response [28]. As part of the health assessments, veterinarians assigned a prognosis for each of the dolphins evaluated. Even 3 years after the DWH spill, dolphins examined in both Barataria Bay and MSS had a high prevalence of guarded or worse prognoses (37% and 35%, respectively), indicating that dolphins from these areas were more likely to be ill, and presumably less likely to survive as compared to dolphins from the healthier reference population in Sarasota Bay, FL, in which only 11% of dolphins had guarded to worse prognoses [28].

While the findings from the health assessment studies suggested that the oil spill and resulting exposure to oil for dolphins would likely result in an impact on population vital rates, in order to estimate the overall injury to each stock, a more quantitative estimate of the effect on vital rates was needed. Follow-up photo-ID monitoring of females determined to be pregnant during the health assessments or from progesterone analysis of remote biopsy samples demonstrated low reproductive success rates; the reproductive success of dolphins from within the heavily oiled BSE waters, including MSS, were less than a third of those reported in other areas not impacted by the spill [29]. The capture-recapture analysis described herein provides for a comparison of post-spill survival rate in MSS to an expected annual survival rate based on previously reported capture-recapture analyses in other BSE sites. It also allows the estimation of dolphin abundance for the MSS portion of the stock. All of these estimates, e.g., survival rates and abundances along with the estimated post-spill reproductive success rates, are essential for the quantification of injury to dolphin stocks. The integration of these estimates into a structured population model that predicts the change and quantifies the associated uncertainty in the post-spill trajectory for northern GoM dolphin stocks is described by Schwacke et al. [30]. In addition to contributing to the quantification of impacts associated with the DWH oil spill, the results described here enhance our knowledge of common bottlenose dolphins in the MSS region, and provide insights for future management decisions.

## Materials and methods

### Study area

MSS is a large, semi-open embayment located in the north-central GoM with a surface area of 2,129 km^2^ (Fig 1a) [31, 32]. East to west, MSS stretches from Cedar Point, AL, to Half Moon Island, LA, and is bordered to the north by Mississippi and Alabama and to the south by Bay Boudreau, LA, and six barrier islands: Dauphin, Petit Bois, Horn, East Ship, West Ship, and Cat [32]. There are large passes between the barrier islands to the GoM, including four dredged channels: Bayou La Batre, Pascagoula/Bayou Casotte, Biloxi, and Gulfport. Average depth at mean low water is 2.98 m, and tides are diurnal with an average range of 0.57 m [32]. Sea surface temperatures range seasonally from approximately 9°C to 32°C [33]. The bottom is soft mud and/or sand [34]. Salinity patterns are complex, varying seasonally with river flow (e.g. Pearl and Pascagoula rivers) and with managed outputs from the Mississippi River, and there are multiple sharp salinity fronts [35, 36]. During minimum low salinities, usually in winter and spring, salinities can be <10 ppt west of Cat Island to Lake Borgne and in eastern MSS adjacent to Mobile Bay, and typically in the 10–20 ppt range elsewhere. During maximum highs, typically during summer and fall, salinities are 15–30 ppt throughout MSS [36–38].

Due to the large size of MSS, logistical reasons (e.g. proximity to the NOAA/NMFS lab in Pascagoula, MS), and historical consistency with prior NMFS studies (e.g. [18, 19, 27]), a subsection of MSS was selected for the study (Fig 1b). The survey area included all MSS waters between the mainland and the barrier islands adjacent to Pascagoula (30.33°N, 88.57°W) and GoM coastal waters to the south of Horn and Petit Bois islands extending 2 km (Fig 1b).

### Capture-recapture photo-ID surveys

Vessel-based, capture-recapture (C-R) photo-ID surveys were conducted in MSS from June 2010 through May 2012 and followed a general robust survey design [39] with eight primary periods completed (Table 1). A complete primary period consisted of finishing all transects (Fig 1b) three times (i.e. 3 secondary occasions) in the shortest time possible and under optimal sighting conditions (Beaufort Sea State ≤3). Cumulatively, all transects totaled ~200 km in length and consisted of two sets of eight east-west lines, spaced ~1 km apart, that generally followed the contour of the islands (Fig 1b). Each secondary occasion (i.e. full coverage of the study area) took two vessels approximately 2 days to complete and was separated by at least 1 day to allow for population mixing (e.g. [40–42]).

**Table 1:** Primary periods (#) and dates of Mississippi Sound common bottlenose dolphin capture-recapture photo-identification surveys (n = 8), and for each, a summary of the number of dolphin groups sighted, individuals sighted and photographed, and number of individuals with unique marks.

Year	Primary period	Dates	Groups	Dolphins	Dolphins photographed	Marked dolphins	Percent marked
2010	1	Jun 23–25, Jul 14–18	94	962	845	686	81%
	2	Oct 14–21	97	931	677	566	84%
2011	3	Mar 14–21	118	981	787	626	79%
	4	May 10–18	87	818	738	578	78%
	5	Jul 12–21	89	1255	936	772	82%
	6	Oct 3–17	147	1194	837	685	82%
2012	7	Jan 5–15	172	1255	1047	837	80%
	8	May 14–21	97	878	779	609	78%

Field protocols used during all DWH NRDA vessel-based photo-ID surveys are described in detail by Melancon et al. [43]. Briefly, surveys were conducted from two, 5–6 m center-console outboard-powered vessels, each crewed by 3–4 researchers following pre-defined transects at approximately 30 km hr^−1^ until a dolphin(s) was sighted. An attempt was made to photograph each dolphin within the group, regardless of dorsal fin distinctiveness, using a Canon EOS digital camera equipped with a 100–400 mm telephoto lens (Canon USA, Inc., Melville, NY, USA). A datasheet was completed for each group sighting, defined as all dolphins in close proximity to one another (i.e. ≤100 m) [44], and included, for example, time, location (via Global Positioning System), water depth, overall group size, number of calves and neonates, environmental conditions, and presence/absence of oil on the surface of the water.

Identifying and matching dorsal fins is critical to obtaining unbiased density and survival estimates given that C-R requires correct identification of individuals within a given region [45–47]. Photo analysis methods are described in Speakman et al. [42] and Melancon et al. [43]. To avoid mismatches, sorted images were graded for photographic quality using a weighted scale that incorporated five characteristics detailed in Urian et al. [48]. Only photographs rated excellent (Q-1) or average (Q-2) were included in subsequent analyses. Sorted photographs were then matched by experienced researchers to the NRDA MSS dolphin dorsal fin catalog using FinBase [49], a customized, Microsoft Access (Microsoft Corporation, Redmond, WA, USA) database. Dorsal fin distinctiveness was based on the extent of dorsal fin markings and was independent of photographic quality. Fins with few markings were considered “unmarked.” Very distinctive fins (D-1; obvious major marks) and average fins (D-2; 2 minor or 1 major mark) were considered “marked” [48]. Sightings (presence/absence and GPS location) of dolphins with distinctive fins (D-1 and D-2) were used in the spatially-explicit robust-design C-R model (described below) and dolphins sighted with unmarked fins were used to calculate the proportion of marked individuals during each primary period.

The estimated proportion of distinctive fins was accounted for in the density estimates by dividing the total calculated density by the proportion of marked fins (Table 1) [50]. If, for example, we estimated 80% of the population were sufficiently marked to allow identification, we inflated the final density of marked individuals by 1/0.8 = 1.25 to account for the unmarked portion of the population. The estimated marked proportion of distinctive fins was estimated from a large number of individual dolphins photographed each session (~800) and uncertainty in this estimate was small (SE ~ [0.8 (0.2) / 800]^1/2^ ~ 0.015). Because uncertainty in the proportion marked was small relative to the other sources of variation, uncertainty due to this factor was not included in density uncertainty estimates.

### Discovery curve and site fidelity

A discovery curve is based on a direct count of new, distinctive individuals identified across survey periods, and has been used to assess population closure and the total number of distinctive animals in a given region [50, 51]. A discovery curve was plotted based upon the total number of unique, “marked” (D-1 and D-2) dolphins across each C-R primary period. Capture histories, a record of whether individuals were photographed during each secondary occasion as well as GPS location and distinctiveness level, were compiled for all “marked” individuals.

To assess short-term (≤2 years) site fidelity, individuals were grouped into three sighting frequency bins based on the total number of primary periods each individual was observed out of eight possible primary periods. Dolphins sighted in 1–2 primary periods were classified as having low site fidelity (LSF) and those sighted in 3–4 primary periods, moderate site fidelity (MSF). High site fidelity (HSF) dolphins were sighted in five or more primary periods. The criteria for the HSF bin was based on the Rosel et al. [52] definition of a resident dolphin as an individual that spends greater than 50% of its time within a given estuary. Comparisons of site fidelity classifications and fluctuations in density/abundance across primary periods may provide insight into different groups or stocks of dolphins that overlap in the MSS region.

### Density, abundance, and survival estimation

Robust-design models assume the sampled population is closed during primary periods, but open between primaries [53, 54]. Under this assumption, robust-design analyses estimate density (or abundance) during and survival between primary photo-ID periods. Following McDonald et al. [15] and Ergon and Gardner [55], we used a robust-design model where the closed portion of the model was replaced by a spatially-explicit C-R model. This model had the advantages of estimating density without a defined study area boundary, improving estimation of capture probability based on photo-ID location and movement data, and separating emigration from death to arrive at a better estimate of survival between primary periods [15]. In addition, a habitat mask and spatial covariate were included. As described by McDonald et al. [15], the sampled area was divided into discrete pixels, each with size 1000 m x 1000 m to define activity centers. The habitat mask restricted movements of activity center locations (derived from individual dolphin photo-ID sighting histories) to water, while the spatial covariate consisted of Island and Inshore strata (Fig 1) (based upon satellite-linked telemetry data discussed in Ranging pattern results below) that allowed separate densities to be estimated in these two areas. The Island stratum was defined as waters <1 km from a barrier island and a 2-km wide corridor in the passes between islands and the Inshore stratum as waters north of the Island stratum and Bay Boudreau. An Offshore stratum (1–2 km offshore of the barrier islands) was also defined as a spatial covariate to include all habitats potentially available to dolphins in the MSS region. However, due to inadequate sampling effort, density estimates in the Offshore stratum were discarded. Abundance estimates for MSS were extrapolated by multiplying the total area of each stratum’s respective region (Island– 242 km^2^; Inshore– 2,196 km^2^) by the stratum densities. Survival between primary periods was estimated by conditioning on the first capture and using the methods of Ergon and Gardner [55] and others [56–59]. Survival was assumed to vary independently between primaries and average survival across all animals in the population at the beginning of the time period was estimated. Survival between primary periods was estimated following McDonald et al. [15] by averaging the equivalent annual survival estimates over the between-primary periods in each year. Uncertainty estimates for summarized survival came from the respective posterior-distributions.

The spatially-explicit robust-design model and its estimation via Bayes Markov Chain Monte Carlo (MCMC) sampling are described in detail in McDonald et al. [15]. Uncertainty in the estimates was quantified by 95% posterior confidence intervals (95% CI). Posterior confidence intervals were computed by discarding the lower and upper 2.5% of the MCMC sample from the posterior distribution of each parameter. The JAGS (http://http://mcmc-jags.sourceforge.net/) and R code (http://cran.r-project.org) necessary to estimate the model, with slight modifications specific to MSS, can be found in the supplementary materials of McDonald et al. [15].

Dolphin abundance for the Mississippi Sound, Lake Borgne, Bay Boudreau Stock as currently defined by NMFS [13] was required for the DWH NRDA dolphin injury quantification [4, 60]. However, only MSS and a small part of extreme northeastern Lake Borgne were included in the dolphin abundance estimates reported here. The rest of Lake Borgne was excluded because it was assumed to be poor dolphin habitat based on average low salinity (i.e. <~8 ppt) [60, 61] (Fig 1a). Bay Boudreau is a shallow marsh complex [62] and dolphin abundance for the NRDA was based on extrapolating the average dolphin density estimate from similar habitat in Barataria Bay [15] to Bay Boudreau [60].

### Satellite-linked transmitter data analysis

**Fig 2:**
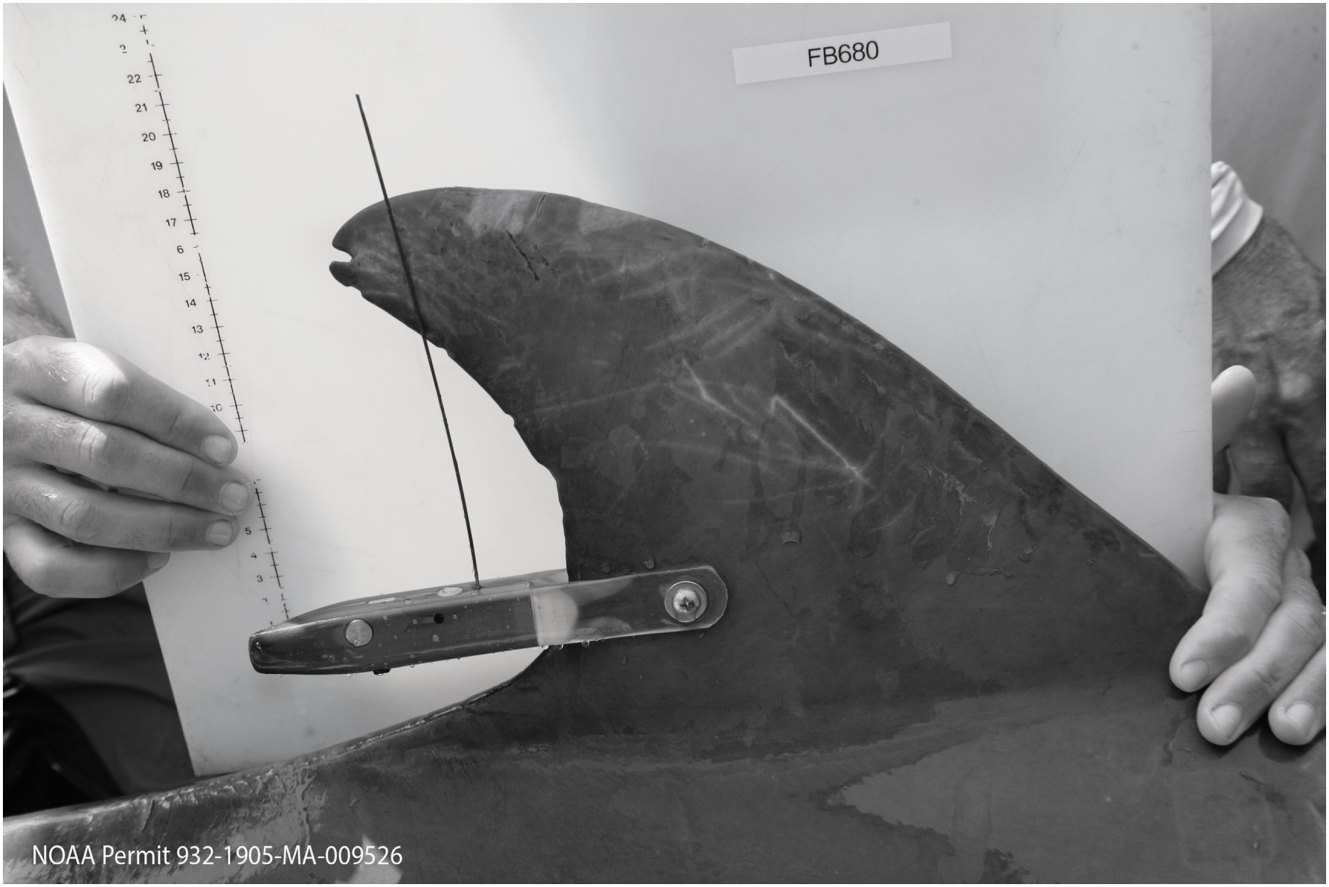
Satellite-linked tag deployed on 19 common bottlenose dolphins in Mississippi Sound during the capture-release health assessment in 2013. SPOT-299B tag (Single-point, Finmount, 2-Lay, Custom; Wildlife Computers, Redmond, WA, USA). Ruler indicates cm. Photo by NOAA.

Dolphins were tagged with SPOT-299B (Single-point, Finmount, 2-Lay, Custom) satellite-linked transmitters (Wildlife Computers, Redmond, WA, USA) (Fig 2) during capture-release health assessments in July-August 2013 (Fig 1b). Protocols for capture-release health assessments can be found in Schwacke et al. [11]. In most cases, a tooth was extracted from each animal under local anesthesia and sectioned to determine age following established protocols [63, 64]. Balmer et al. [65] and Wells et al. [12] discuss in detail tag programming, tag attachment, tag transmission rates, and modes of tag failure.

Satellite-linked locations were received and processed by the Argos Data Collection and Location System. Argos classifies location quality relative to an estimated error radius. The best quality data, LC3, has an estimated error of <250 m. LC2 has an estimated error of <500 m. LC1 locations are estimated to be accurate to within 1,500 m. Only LC3 and LC2 locations were used for analyses.

#### Location data and home range analyses

Each individual’s ranging pattern was described as overall home [95% utilization distribution (UD)] and core (50% UD) ranging areas, calculated using a kernel interpolation method while accounting for shoreline barriers. The UDs measure areas of intense use; therefore, the resulting ranging areas may not be continuous, but rather broken in space [66]. Home range size for dolphins in BSEs have been found to vary by sex (females usually have smaller home ranges) and the numbers of locations used to define the ranges [67]. Urian et al. [67] determined that fixed kernel home range sizes for dolphins in bays on the west coast of Florida changed little with >150 locations, based on sighting data. For MSS tag location data, a lower threshold of 150 locations was established for statistical analyses; however, all animals received a home and core range calculation. To reduce the potential for autocorrelation, one randomly selected location per day was retained for home range analysis. Home and core range calculations are sensitive to a smoothing parameter (*h*) or bandwidth that determines the size and shape of spatial use [68, 69]. A rule-based *ad hoc* method was applied to estimate the appropriate smoothing parameter for each individual’s home range [69–71]. Analysis of estimated smoothing parameters was completed using the Home Range Tools for ArcGIS extension for ArcGIS 9.0 (ESRI, Redlands, CA, USA). Analyses for kernel interpolation while accounting for barriers were completed using the Geostatistical Analyst and Spatial Analyst toolboxes in ArcGIS 10x, following methods suggested by MacLeod [72]. Any remaining land areas were subtracted from the final home and core range areas to ensure resulting range areas represented only water. Three parameters were used to describe home range size: (1) overall home range (95% UD), (2) core area (50% UD), and (3) largest dimension of the range (longest distance between 2 locations).

#### Hot spot analysis

A hot spot analysis adapted from Smith et al. [73] was applied to examine clustering patterns over a grid of monthly summed locations to assess shifts in tagged animals’ ranges over time. This analysis used the Getis-Ord Gi* statistic [74] to calculate a Z-score (Gi* statistic) and p-value for each grid cell based on the local sum of locations compared to a global summation across the entire study area (reviewed in [73]). If the Z-score of the local sum was significantly different (both p < 0.05 and p < 0.01 were assessed) from the global sum, then the data contained either hot spots or cold spots (i.e. clusters of high value grid cells or low value grid cells, respectively) [75].

Satellite-linked tag locations were summed by month per grid cell with 1 km^2^ resolution (Smith et al. 2013) to create the grid for hot spot analysis in ArcGIS 10x. This resolution was chosen to account for the <500 m estimated error of LC2 tag locations. The time period for data collection ranged from August 2013 through December 2013, the period during which the majority of tagged dolphins were transmitting. Two dolphins, both tagged on the south side of Horn Island, were excluded from hot spot analyses. Tracking data indicated these dolphins had ranges exclusively around the barrier islands and that they might belong to a separate “community” from those tagged further north, where a community of dolphins within a stock is an assemblage of interacting dolphins but not a demographically independent unit (see [67]). The limited sample size for these two individuals prevented a hot spot analysis for this hypothesized community.

Community spatial autocorrelation was assessed using the “Incremental Spatial Autocorrelation” (ISA) tool, which conducts a series of Global Moran’s I statistics across different distance intervals. The initial distance interval to run the ISA was the “Average Nearest Neighbor” distance. The distance threshold at which community spatial autocorrelation initially peaked was selected as the distance threshold for the hot spot analysis because this distance represents the scale at which similar values of satellite-linked tag locations/grid cells are most clustered together [73, 75]. A spatial weights matrix file was customized for each monthly hot spot analysis which specified the distance threshold for the hot spot analysis as well as the number of neighbors to be calculated in the local sum. Eight neighboring grid cells were included in every local sum calculation for each grid cell. If the distance threshold did not contain eight neighboring cells (i.e. perimeter cells) then the distance threshold was extended to include eight neighboring grid cells. Hot spots were retained at p < 0.01 and p < 0.05 to examine trends in clustering patterns. A Bonferroni correction has been suggested to control for multiple comparisons between grid cells but this correction can result in highly conservative p-values for large sample sizes [74]. This correction would result in p-values that range between 0.00010 and 0.00012 and these p-values were determined to be too strict for this exploratory analysis. The hot spot results from two consecutive months were examined for overlap to determine short-term seasonal shift. The “Intersect” tool was used to determine overlap between monthly hot spots. The number of overlapping hot spots between comparison months was divided by the number of total hot spots for the two comparison months. This value provided a percentage of overlap between comparison months. In total, four comparisons were made to examine hot spot overlap between each possible two-consecutive-month period from August to December.

Research was conducted under National Marine Fisheries Service Scientific Research Permit Nos. 932-1905/MA-009526 (capture-release activities and follow-up monitoring of animals) and 779–1633 (photo-ID surveys). All research protocols used were approved by a NOAA Institutional Animal Care and Use Committee.

## Results

### Discovery curve and site fidelity

From June 2010 through May 2012, 116 photo-ID surveys with two vessels, totaling 60 survey days, were conducted in MSS to complete eight C-R primary periods (Table 1). During the two-year project, 13,239 km were surveyed during 820 hrs on the water (n = 256 hrs in contact with dolphins). Over 72,000 photographs were taken of 903 dolphin groups. Mean group size was 10 (range 1–80) and varied seasonally with lowest and highest mean group size in the winter ($\bar{x}$ = 7.4, SE = 0.52, n = 172) and summer ($\bar{x}$ = 14.5, SE = 1.40, n = 89), respectively.

**Fig 3:**
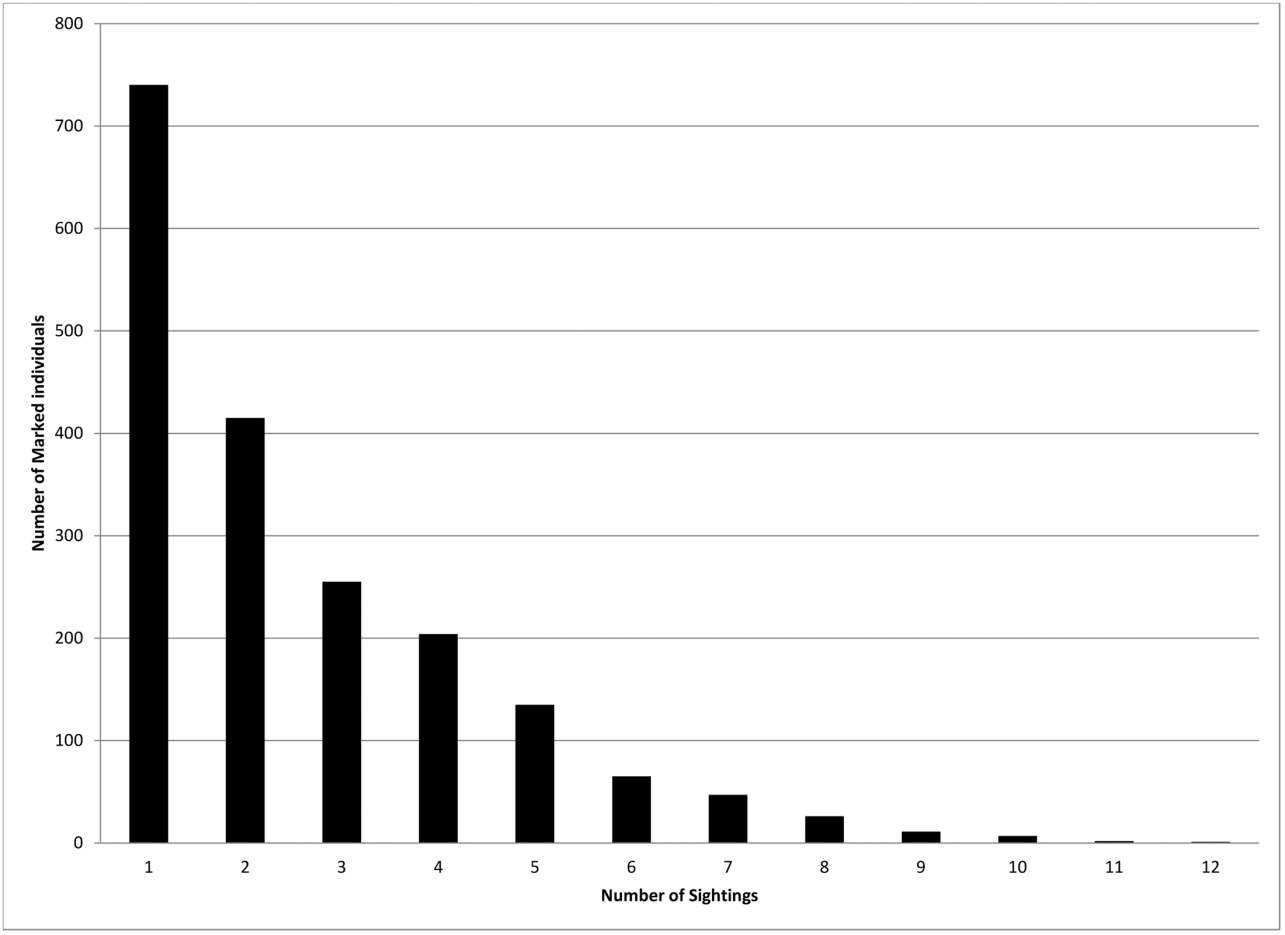
Sighting frequency of marked individual common bottlenose dolphins from Mississippi Sound photo-identification surveys during 2010–2012.

During the C-R study, 1,908 distinctively marked individuals were identified with 654 calves and 91 neonates recorded. Unlike distinctive individuals that can be identified across surveys, the majority of calves and neonates were not distinctive; thus, these total counts likely include resightings of the same individuals. The vast majority of neonates (97%) were sighted in March or May. The calculated marked proportion of individuals varied slightly across primary periods (78–84%) (Table 1). Of the 1,908 distinct individuals, 39% (n = 740) were only sighted once (Fig 3).

**Fig 4:**
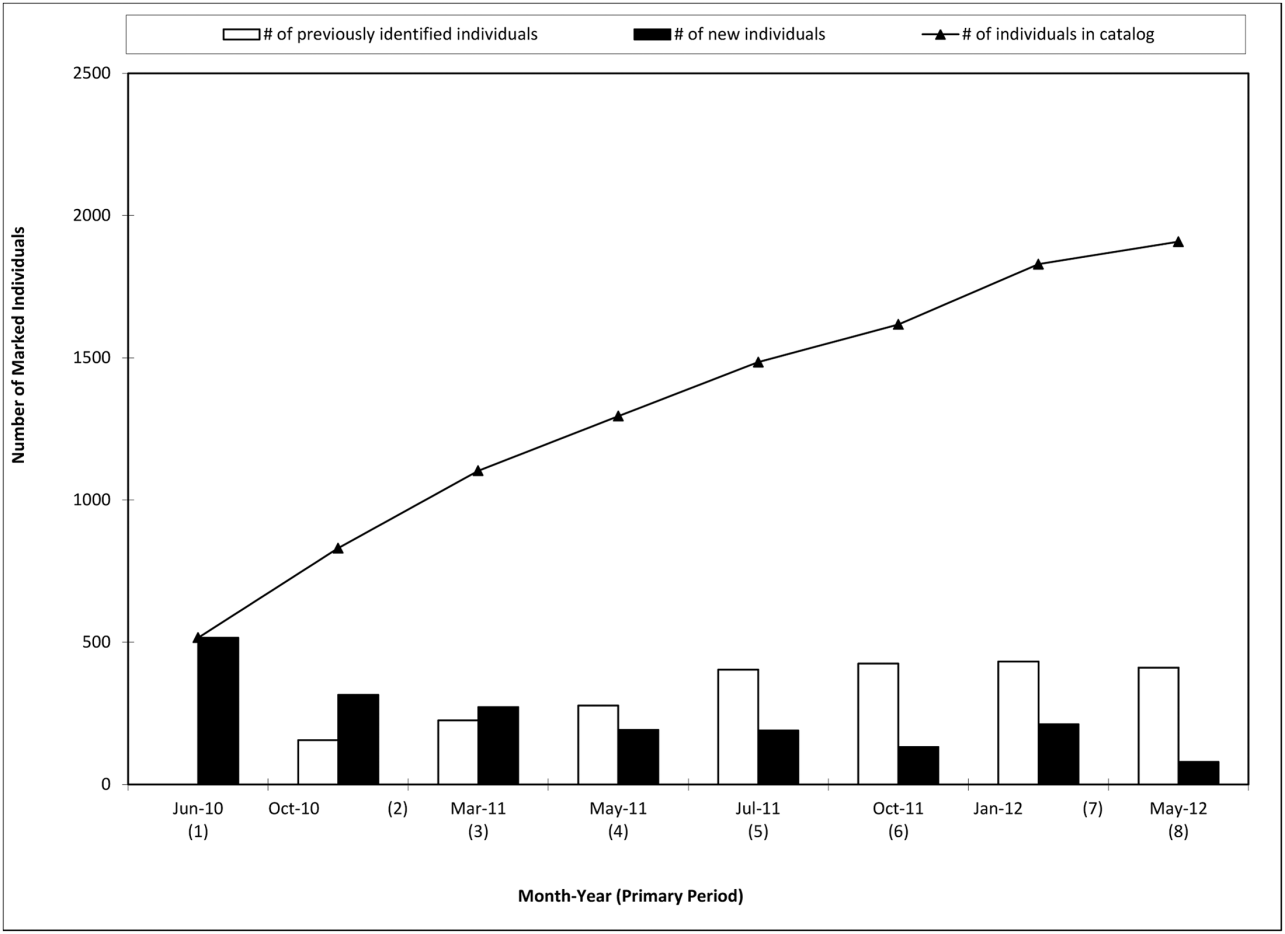
Number of individuals sighted during photo-identification surveys and discovery curve for common bottlenose dolphins in Mississippi Sound during 2010–2012.

With the exception of January 2012, the detection of new individuals decreased as the two-year study continued, while the number of previously sighted dolphins remained constant during the last year of the study (Fig 4). The detection rate increased steeply through January 2012, followed by a more gradual climb until May 2012, when the curve is more flat exhibiting just a slight increase at the final primary period (Fig 4).

**Fig 5:**
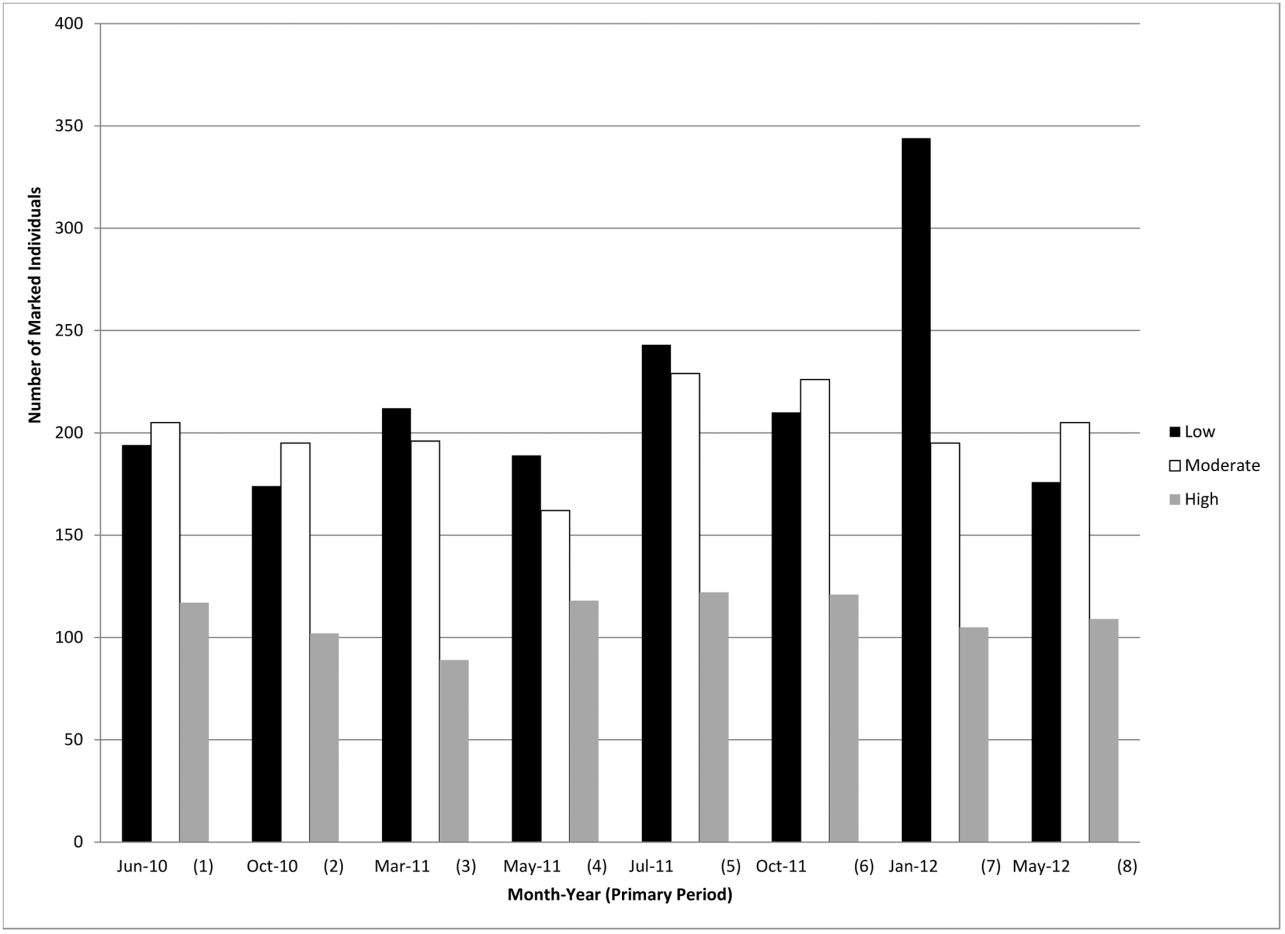
Number of marked common bottlenose dolphin individuals from each site fidelity bin observed during photo-identification surveys in Mississippi Sound during 2010–2012 (Low = 1–2 primary periods; moderate = 3–4 primary periods; high = ≥ 5 primary periods).

The majority of individuals (67%; n = 1,274) were classified as LSF (sighted in 1–2 primary periods) while 8% (n = 162) were sighted in five or more primaries (HSF). LSF and HSF peaked in January 2012 and July 2011, respectively, whereas the fewest number of HSF were seen in March 2011 (Fig 5).

### Density and abundance

**Fig 6:**
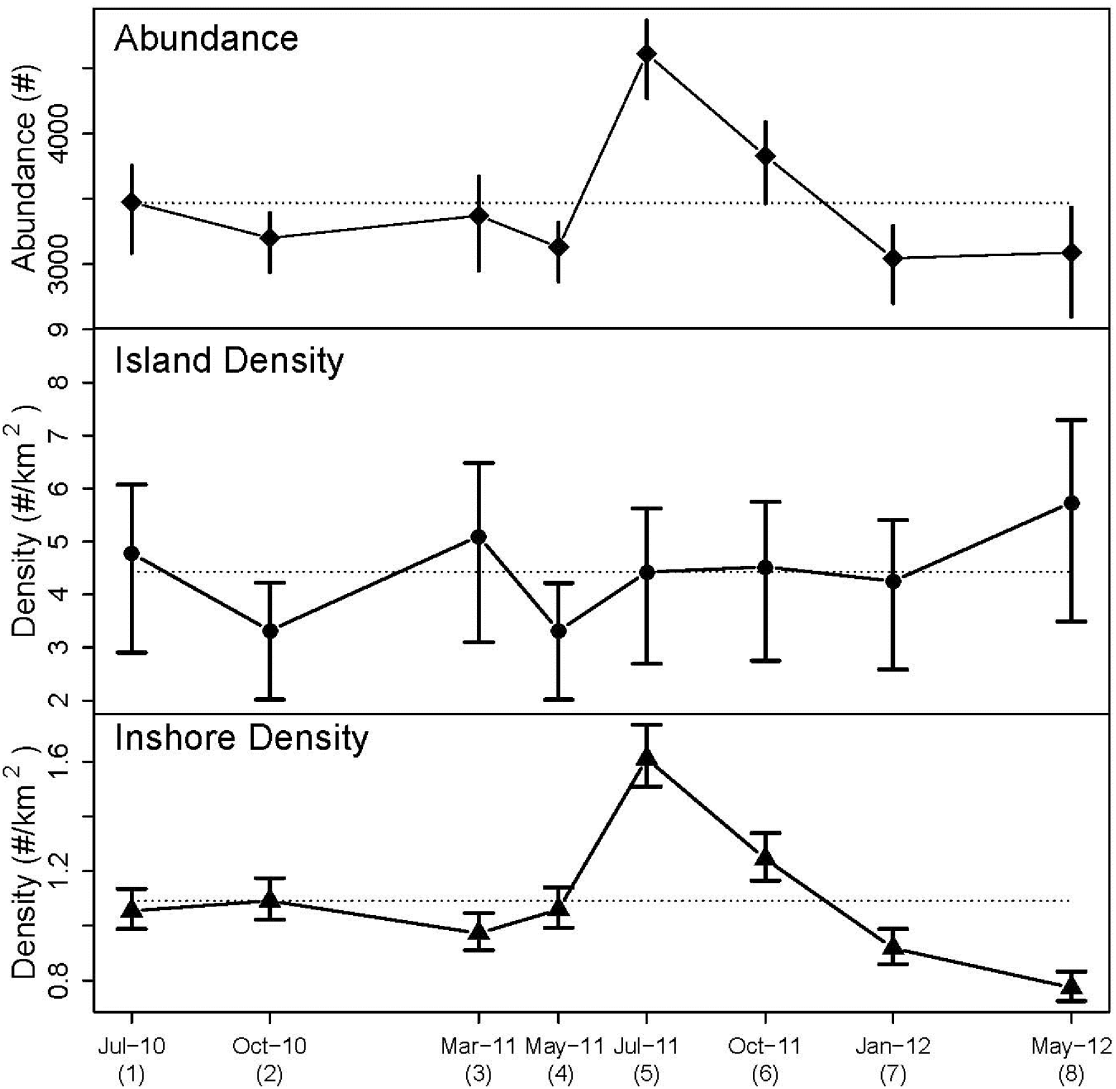
Overall common bottlenose dolphin abundance and density estimates by stratum in Mississippi Sound from the spatially-explicit robust-design capture-recapture model applied to 2010–2012 photo-identification data.

MSS dolphin density differed between strata (Table 2, Fig 6). Overall, the mean Island density was approximately 4.4 times greater than that for the Inshore stratum. Mean Inshore density was 1.09 dolphins km^−2^ (95% CI: 1.02–1.17) with point estimates ranging from 0.77 to 1.61 dolphins km^−2^ across all primary periods. Inshore densities were approximately constant from July 2010 through May 2011, were elevated in July and October 2011, and then decreased during January and May 2012. Mean Island stratum density was 4.43 dolphins km^−2^ (95% CI: 2.70–5.63) with primary period point estimates ranging from 3.32 to 5.74 dolphins km^−2^. Precision of the Island stratum estimates as measured by width of the 95% posterior intervals was less than the precision of the Inshore stratum estimates. Island densities were relatively constant from 2010–2012 with all primary periods having overlapping confidence intervals. Mean MSS density and abundance was 1.42 dolphins km^−2^ (95% CI: 1.28–1.53) and 3,469 dolphins (95% CI: 3,113–3,725), respectively, and point estimates for primary periods ranged from 1.89 dolphins km^−2^ and 4,610 dolphins (July 2011) to 1.25 dolphins km^−2^ and 3,046 dolphins (January 2012). MSS density and abundance were generally comparable across all primary periods except during July 2011 in which density and abundance were at the highest levels observed in this study (Table 2, Fig 6).

**Table 2:** Estimated common bottlenose dolphin density (dolphins km^−2^) and abundance (dolphins) during primary photo-identification capture-recapture periods, and averaged over the study period in Mississippi Sound (MSS).

		Island Density	Inshore Density	MSS Density	MSS Abundance
Period	Date	Est (low, high)	Est (low, high)	Est (low, high)	Est (low, high)
1	Jul 2010	4.78 (2.91, 6.07)	1.05 (0.99, 1.14)	1.42 (1.27, 1.54)	3474 (3088, 3752)
2	Oct 2010	3.32 (2.02, 4.22)	1.09 (1.02, 1.17)	1.31 (1.21, 1.39)	3201 (2942, 3391)
3	Mar 2011	5.10 (3.10, 6.48)	0.97 (0.91, 1.05)	1.38 (1.21, 1.51)	3372 (2951, 3672)
4	May 2011	3.32 (2.02, 4.22)	1.06 (0.99, 1.14)	1.28 (1.18, 1.36)	3130 (2871, 3320)
5	Jul 2011	4.42 (2.69, 5.62)	1.61 (1.51, 1.73)	1.89 (1.75, 2.00)	4610 (4271, 4865)
6	Oct 2011	4.52 (2.75, 5.75)	1.24 (1.17, 1.34)	1.57 (1.42, 1.68)	3828 (3467, 4086)
7	Jan 2012	4.25 (2.59, 5.40)	0.92 (0.86, 0.99)	1.25 (1.11, 1.35)	3046 (2702, 3293)
8	May 2012	5.74 (3.49, 7.29)	0.77 (0.72, 0.83)	1.27 (1.07, 1.41)	3089 (2600, 3435)
**Mean Density**		**4.43**	**1.09**	**1.42**	
		**(2.70, 5.63)**	**(1.02, 1.17)**	**(1.28, 1.53)**	
**SD (Density)**		**1.101**	**0.042**	**0.081**	
**Mean Abundance**		**1074**	**2395**		**3469**
		**(653, 1365)**	**(2244, 2578)**		**(3113, 3725)**
**SD (Abundance)**		**266.9**	**92.2**		**197.7**

-Abundances were calculated by expanding densities to size of the strata in all of MSS (2438 km^2^: Island, 242 km^2^ and Inshore, 2196 km^2^)

-“low, high” = 95% posterior confidence interval

-SD = standard deviation

Vertical bars are approximate 95% posterior confidence intervals. Symbols are plotted at mid-point dates of primary sampling periods. Dashed horizontal lines are temporal averages of their respective time series. Note differences in y-axis scales.

### Survival

Seven inter-primary survival rates were estimated that ranged from 0.31 (95% CI: 0.23–0.40) to 0.81 (95% CI: 0.73–0.89) (Table 3). The survival rate of marked dolphins in the first year post-spill (10 July 2010–16 July 2011) was estimated as 0.73 (95% CI: 0.67–0.78). The survival rate for the remaining periods was adjusted to produce an annual survival rate that would be comparable to published rates. Survival during the following 10-months (16 July 2011–17 May 2012), annualized to 1-year, was estimated as 0.52. However, this survival rate spans the last two capture occasions and therefore may be partially confounded with capture probability [76]. Furthermore, this partial year period did not sample during mid-May to mid-July 2012, a period that generally has lower stranding rates as compared to the early spring months in which surveys were conducted. The sampling period therefore may have negatively biased the survival estimate. As this estimate was thought to be potentially biased and thus unreliable, the final inter-primary period (10 January 2012–17 May 2012) was dropped for the analysis of survival rate. After dropping the final inter-primary period and annualizing to 1-year, the result produced a survival estimate of 0.78 (95% CI: 0.74–0.81), and was used as an estimate of survival for the second year post-spill.

**Table 3:** Estimated common bottlenose dolphin annualized (or ‘equivalent annual’) survival probabilities for Mississippi Sound by primary period (See text: yr1 = 10 July 2010 to 16 July 2011, yr2 = 16 July 2011 to 09 January 2012).

Primary period	Date	Estimated survival	95% CI		Annual interval
			Lower	Upper	
1	8/29/2010	0.723	0.602	0.859	yr1
2	1/1/2011	0.812	0.729	0.894	yr1
3	4/15/2011	0.561	0.435	0.683	yr1
4	6/14/2011	0.745	0.553	0.919	yr1
5	8/28/2011	0.52	0.432	0.611	yr2
6	11/24/2011	0.686	0.57	0.82	yr2
7	3/14/2012	0.314	0.236	0.395	yr2

### Ranging patterns

**Fig 7:**
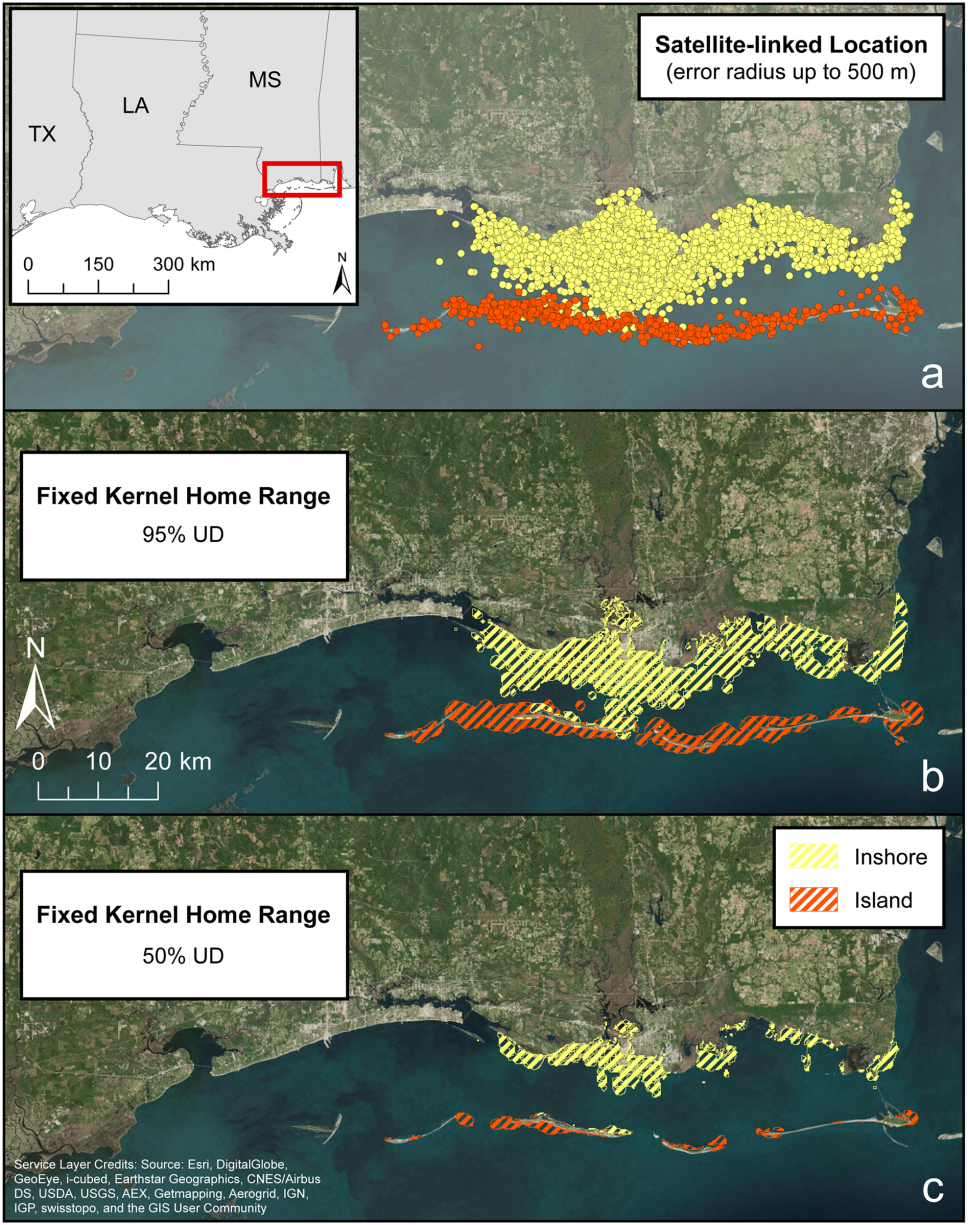
Locations and ranging patterns from common bottlenose dolphins satellite tagged in Mississippi Sound during July 2013–April 2014. a. Location Class 2 and 3 satellite-linked locations from each dolphin. b. Composite fixed kernel 95% utilization distribution (UD) home range. c. 50% UD core range.

Satellite-linked tags were deployed on 19 dolphins– 8 females and 11 males (Table 4, Fig 1b). A tooth for aging was not collected for pregnant females (n = 7) or early releases (n = 1; FB “freeze brand” 669, a female), therefore no females were aged. Tags transmitted from 73 to 253 days, with a mean of 491 locations (range = 222–710) (Table 4). All high quality location data (LC3 and LC2) indicated that tagged dolphins remained in MSS and adjacent waters throughout the tracking period (Fig 7). Two distinct ranging patterns emerged from the telemetry data that were used to categorize tagged dolphins: (1) inshore dolphins used waters primarily in the northern half of MSS (n = 17) and (2) island dolphins ranged primarily along a narrow corridor associated with the barrier islands that included both the north and south sides (n = 2) (Fig 7). These ranging patterns were also used as the rationale for defining Inshore and Island strata for estimating dolphin density. However, the overall ranges of two inshore dolphins (FB 667 and 682), and the two island dolphins did include waters of the Island stratum, and waters of the Inshore and Offshore strata, respectively. Two individuals (FB 661 and 665) tagged in Grand Bay (Fig 1b) ranged eastward into extreme eastern MSS and western Mobile Bay.

**Table 4:** Mississippi Sound common bottlenose dolphins satellite-linked tagged during summer 2013 and grouped by stratum (Inshore or Island) (ND = not determined; SD = standard deviation).

Freeze Brand No.	Deploy Date	Age (yrs)	Length (cm)	Tag Life (days)	Fixed Kernel Home Range Area (km^2^)		Maximum Distance Between Locations (km)
					95%	50%	
*Inshore—Females (n = 8)*							
661	23-Jul	ND	228	158	101.20	21.76	31.83
665	23-Jul	ND	231	218	210.96	48.16	42.28
667	24-Jul	ND	230	233	166.60	30.64	36.89
669	30-Jul	ND	177	253	101.80	27.44	25.26
671	30-Jul	ND	236	175	87.56	23.56	29.22
673	30-Jul	ND	229	203	120.72	27.40	28.09
675	31-Jul	ND	235	157	62.80	15.00	16.43
677	31-Jul	ND	231	213	44.84	8.92	19.9
**Mean**		ND	**224.6**	**201.3**	**112.1**	**25.4**	**28.7**
**SD**		ND	**19.44**	**35.1**	**54.25**	**11.65**	**8.47**
*Inshore—Males (n = 9)*							
660	25-Jul	20	264	228	164.36	28.52	33.23
662	25-Jul	5.5	233	189	61.60	12.88	17.11
664	26-Jul	8.5	234	171	88.48	27.36	24.99
668	26-Jul	5.5	213	164	56.84	15.44	23.96
670	26-Jul	5.5	211	228	95.36	23.12	23.59
672	28-Jul	7	202	252	48.80	10.92	18.21
678	30-Jul	30	265	150	129.32	36.08	34.09
680	1-Aug	6.5	208	156	26.24	7.20	14.32
682	1-Aug	15	244	188	131.08	27.92	23.08
**Mean**		**11.5**	**230.4**	**191.8**	**89.1**	**21.1**	**23.6**
**SD**		**8.58**	**23.77**	**36.23**	**45.35**	**9.78**	**6.73**
*Island—Males (n = 2)*							
674	29-Jul	19	260	73	125.2	18.64	79.29
676	29-Jul	15	241	165	190.52	36.88	69.52
**Mean**		**17**	**250.5**	**119**	**157.9**	**27.8**	**74.4**

The mean 50% and 95% UDs and maximum distance between locations were generally similar for inshore males and females. The two island dolphins had mean 50% and 95% UDs comparable to inshore dolphins (Table 4). However, the maximum distance between locations for the island dolphins was approximately 3 times greater than that of the inshore dolphins.

**Fig 8:**
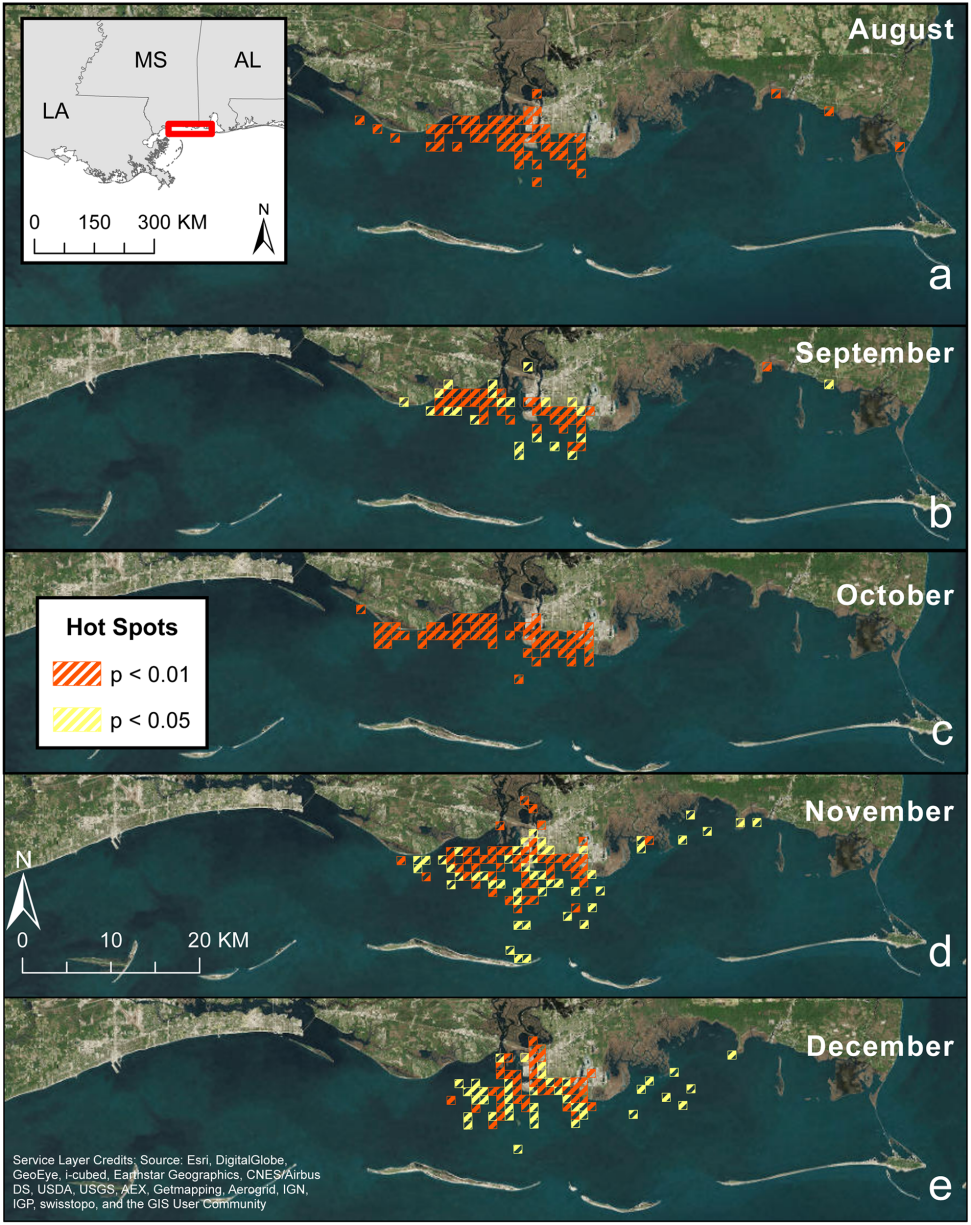
Monthly 2013 Getis-Ord Gi* (“hotspots”) of the inshore common bottlenose dolphins tagged with satellite-linked transmitters in Mississippi Sound.

Monthly hot spot analyses of inshore dolphins identified significant clustering (at the p < 0.05 and p < 0.01 levels) of tagged dolphins for August through December 2013 (Fig 8). During August through October, hot spots were concentrated along the mainland inshore waters in and near Pascagoula Bay (i.e. Bellefontaine Point to Point aux Chenes) (Figs 1b & 8). During November and December, hot spots extended somewhat to the south. The hot spots east of Point Aux Chenes represent the two dolphins tagged in Grand Bay (FB 661 and 665). The number of hot spots at p < 0.05 were generally comparable in August, September, and October, increased during November, and then decreased in December (Table 5). The p < 0.05 and p < 0.01 hot spots, during August and October, were generally comparable, while in September, November, and December, there were fewer p < 0.01 hot spots identified. The percentage of hot spot overlap decreased over the 5-month period (Table 5).

**Table 5:** Monthly hot spot number (p < 0.05 and p < 0.01), and hot spot overlap number and percentage between consecutive months at p < 0.05.

**Tracking Month**	**Hot Spot Number (p < 0.05)**	**Hot Spot Number (p < 0.01)**	**Hot Spot Overlap (number and percentage at p < 0.05)**			
			August	September	October	November
August	60	60				
September	58	36	32(37.2%)			
October	62	62		30(33.3%)		
November	100	53			34(26.6%)	
December	81	41				39(27.5%)

## Discussion

### Density and abundance

McDonald et al. [15] estimated dolphin densities for two Inshore strata (East and West) and one Island stratum for Barataria Bay with strata definition similar to those used for MSS. While we estimated the overall Island dolphin density to be about four times larger than that for the Inshore stratum, McDonald et al. [15] estimated the Island density in Barataria Bay to be at least 10 times larger than densities for the Inshore strata. The passes between barrier islands are probably one of the major reasons, among others [15, 77], for the increased dolphin densities for island strata. Other researchers have noted the tendency of dolphins to concentrate near entrances to estuaries and lagoons which likely act as funnels and concentrate fish moving between ocean and inshore waters (e.g. [77–79]).

Previous studies in MSS primarily estimated dolphin density and abundance using aerial- and boat-based line transect surveys (reviewed in [13]) (Table 6). In general, while differences in methods, years, seasons and areas surveyed may confound direct comparisons, densities derived from boat-based line-transect (BBLT) surveys were much larger than those from aerial line-transect surveys (Table 6). Aerial survey estimates, prior to 2012, have severe negative bias because they did not attempt to correct for perception and/or availability biases [“g(0)”] primarily related to platform speed [80–82]. While not quantified, these biases are most likely a result of relatively small average group sizes and water turbidity in MSS. That is, entire groups are probably missed because all the members are subsurface and are not seen from fast moving aircraft because of turbidity, and those probabilities and g(0) are extremely difficult to accurately estimate in MSS. BBLT surveys in MSS are conducted at speeds ~5 times slower than aerial surveys and consequently seem to be much less negatively biased.

**Table 6:** Review of common bottlenose dolphin density (dolphins km^−2^) estimates for the Mississippi Sound region grouped by survey type, source, years of study, and season (Coefficients of variation are in parentheses).

Survey Type	Source (area surveyed)	Years of study	Summer	Fall	Winter	Spring
**Capture-recapture**	Present study (west of Horn Island^1^ to east of Petit Bois Island)	2010	1.4(0.06)	1.3(0.04)		
		2011	1.9(0.04)	1.6(0.05)		1.3(0.04)
		2012			1.2(0.06)	1.3(0.10)
**Boat-based line-transect**	Pitchford et al. 2016a (MS-AL border through Lake Borgne)	2011–2012			0.7(0.25)	1.1(0.22)
		2012–2013	0.9(0.26)	0.5(0.23)	0.8(0.28)	0.3(0.31)
		2013	0.7(0.23)	0.8(0.25)		
	Miller et al. 2013^2^ (west of Cat Island to west of Horn Island)	2007–2008	1.5(0.11)		0.9(0.16)	
	Hubard et al. 2004 (west of Horn Island to east of Petit Bois Island)	1995	1.3(0.17)	0.6(0.23)	0.6(0.28)	
		1996	1.2(0.29)			0.8(0.29)
	Mullin & Hoggard 1992 a, b (west of Cat Island to CedarPoint, AL)	1991	1.1(0.20)		0.5(0.25)	
		1992	1.1(0.16)		0.6(0.26)	
	Lohoefener et al. 1990a (Half Moon Island to Cedar Point, AL)	1984–1985	1.3 (0.31)	1.0 (0.18)	0.3 (0.13)	0.7 (0.18)
	Lohoefener et al. 1990a (west of Cat Island to east of Petit Bois Island)	1985–1986	1.5 (0.37)	1.0 (0.28)	0.4 (0.13)	1.0 (0.21)
**Aerial line-transect**^3^	Waring et al. 2015^3^	2012	0.5 (0.59)	0.3 (0.41)	0.2 (0.63)	0.7 (0.42)
	Blaylock & Hoggard 1994^3^	1992		0.2 (0.49)		
	Scott et al. 1989	1985–1986	0.5 (0.16)	0.1 (0.18)	0.1 (0.13)	

^1^ –Geographic locations referenced are shown in Fig 1.

^2^ –Densities calculated from the coastal and island strata in Table 3 from Miller et al (2013).

^3^ − Aerial surveys covered the Mississippi Sound, Lake Borgne, Bay Boudreau stock area and densities were calculated by dividing abundance by stock area.

In most cases, surveys of all types, including ours, have determined that dolphin density in MSS is higher in summer relative to winter (Table 6). It has been suggested that some coastal dolphins inhabit BSE waters on a seasonal basis [19, 40]. One hypothesis for these seasonal fluctuations in density is increased prey abundance within MSS during summer, and prey species shifting movements inshore [19, 23]. Large numbers of dolphins could also be moving into MSS to feed on prey stirred up by shrimp trawling and discarded bycatch [83] during shrimp season beginning in the early summer. The lower dolphin density we estimated from summer 2010 (July) compared to summer 2011 (July) may have been a result of the partial or complete shrimp fishery closure in MSS due to the oil spill [84] or shrimp trawlers being used for the more lucrative oil spill cleanup operations. Additionally, it is possible that impacts from the DWH spill, either directly through increased mortality of dolphins or indirectly through other ecosystem perturbations, disturbed typical seasonal movements of dolphins in the MSS region. Continued research will be necessary to monitor dolphin movements in the MSS region and interactions with dolphins from adjacent regions as these dolphin stocks recover over time following the DWH disaster.

Despite the numerous dolphin BBLT studies in MSS, it is difficult to develop a comprehensive picture of density and abundance for all of MSS because the variety of study areas potentially confounds the direct comparison of results (Table 6). Lohoefener et al. [24] conducted the only BBLT survey that surveyed all of MSS from Half Moon Island, LA, to Cedar Point, AL. In the remainder of the studies, different subareas were surveyed that, in some cases, included the Gulf side of the barrier islands to various distances [20, 23] or included Lake Borgne [20], which further confounds comparisons. Most BBLT surveys were conducted in all or part of the area from west of Cat Island to east of Petit Bois Island. The MSS areas from Cat Island west to Lake Borgne and extreme eastern MSS adjacent to Mobile Bay have received less attention, and due to the influence of river outflow, have the largest seasonal range of salinity, which may influence dolphin abundance. Nevertheless, with the exception of the overall seasonal estimates by Pitchford et al. [20], dolphin densities throughout MSS have been similar for summer (1.1–1.5 dolphins km^-2^). The winter estimate by Miller et al. [23] may be higher (0.9 dolphins km^-2^) because their study area included GoM waters, but otherwise winter densities for MSS from Cat Island east were similar (0.4–0.6 dolphins km^-2^). Pitchford et al. [20] estimated dolphin densities for seven east-west strata blocks from the Mississippi-Alabama border through Lake Borgne. Given the low precision of the stratum estimates, the seasonal dolphin densities for each stratum appear generally similar across MSS except for the lower densities in winter for the area west of Cat Island (~27% of MSS). The consistency of year-to-year density reduction in western MSS will require further study. For example, during remote biopsy sampling in January 2013, 20 dolphin groups ($\bar{x}$ = 4.6 dolphins/group, SE = 0.91, range 1–18) were sighted in a broad area west of Cat Island in the northern half of MSS (NMFS unpublished) in an area where Pitchford et al. [20] recorded few dolphin groups. Thus, it is currently unclear what cues may be influencing dolphin density and distribution in western MSS.

The MSS seasonal C-R density estimates during 2010–2012 (range: 1.25–1.89 dolphins km^−2^) were generally larger than those from BBLT. In constructing C-R-based abundance estimates for MSS (Table 2), the densities from the Island stratum that included 1 km of Gulf waters and the Inshore stratum were extrapolated well beyond the boundaries of the study area to include all of MSS and extreme eastern Lake Borgne. Due to the larger densities and the extrapolation to all of MSS, seasonal abundances (3,046–4,610 dolphins) were larger than those previously estimated. The only recent estimates for the entire MSS were from four seasonal aerial surveys in 2012 that include Lake Borgne and Bay Boudreau and included a partial correction for g(0) resulting in estimates ranging from 2,395 (spring) to 900 (winter) [13]. Miller et al. [23] using BBLT surveys estimated 2,073 and 1,184 dolphins for summer and winter, respectively, for a study area that covered ~55% of MSS and included a stratum with adjacent GoM waters. Seasonal BBLT survey estimates by Pitchford et al. [20] that also included the GoM ranged from 738 to 3,236 dolphins for MSS.

Well-designed MSS-wide BBLT or C-R surveys need to be conducted to get the best information on MSS dolphin abundance but logistically it would be quite challenging, requiring multiple vessels across the same survey period as has been conducted in estuarine/nearshore waters of North Carolina [45, 48]. Given the lack of a recent MSS-wide survey, the extrapolation of the C-R densities to all of MSS, while not ideal, provides the most accurate estimates of dolphin abundance available for MSS. The C-R method for estimating density appears to be superior to the line-transect method as conducted to date. BBLT surveys in MSS are probably negatively biased because transects have been cut short near the Island stratum, where we estimated the highest densities of dolphins, and the mainland due to navigational hazards (e.g. shallow water and sand bars) [19, 24, 85, 86]. The east-west oriented transect lines surveyed by Pitchford et al. [20] did not appear to survey the C-R Island stratum or cross a potential mainland-to-island (i.e., north-south) dolphin density gradient and may not have been optimal for unbiased estimates of absolute density. The C-R abundance estimates may be positively biased in winter to some unknown degree but overall the C-R density estimates are less biased than those from small boat and aerial line transect surveys. Given that previous BBLT studies have indicated that dolphin densities throughout most of MSS are similar, the extrapolated abundance estimates more accurately reflect the number of dolphins inhabiting MSS.

### Survival

Estimated survival rates for MSS dolphins were low in the post-spill period July 2010 –January 2012. The estimated rate in the first year post-spill (July 2010 –July 2011) was only 0.73; the rate for the second period (July 2011 –January 2012), adjusted as an annual rate, was only 0.78. In comparison, a C-R study of dolphins near Charleston, SC, reported an annual survival rate of 0.95 (95% CI: 0.88–1.00) [42], and a long-term photo-ID study in Sarasota Bay, FL, reported an annual 0.96 survival rate [87]. The comparatively low survival for MSS dolphins is not surprising given the overlap of the survey period with a cluster of increased dolphin strandings reported for Mississippi as part of the broader cetacean Unusual Mortality Event declared for the northern GoM [88]. In fact, 2011 was identified as a “Large Scale Mortality Year” for Mississippi, defined as a year in which the total number of dolphin strandings met or exceeded the 95^th^ quantile calculated from the prior baseline years for the state. Unfortunately, our photo-ID surveys did not continue past May 2012, therefore we cannot confirm that survival increased in the latter part of 2012 as the stranding rates in Mississippi reportedly declined [88].

The survival rates in MSS were also lower than those estimated for dolphins from heavily oiled Barataria Bay (annual survival in Barataria Bay had a range of 0.83–0.85 over the approximate same period; see [15]). The cumulative oiling index calculated for Barataria Bay was higher than the index for MSS, although the index for Chandeleur Sound, which is between the two regions, was higher than either [60]. While the cumulative oiling index is informative as a relative measure of oiling among regions, there are multiple scenarios for route (e.g. inhalation, aspiration, ingestion, dermal) and timing of oil exposure for dolphins across the various regions and habitats within the DWH oil spill footprint; therefore, we cannot definitively say that Barataria Bay dolphins were significantly more exposed than dolphins in MSS. It is also possible that pre-existing stressors for MSS dolphins increased their susceptibility to oiling effects, and exacerbated adverse health outcomes and mortality following the DWH spill. For example, an increased seroprevalence of morbillivirus titers was found in MSS dolphins relative to dolphins sampled in Barataria Bay or other parts of the northern GoM [89], suggesting a possible historically higher exposure to morbillivirus for MSS dolphins. However, the same study reported that morbillivirus was detected by PCR (polymerase chain reaction) in only 10% of stranded cetaceans tested during 2010–2014, indicating that it was not a major contributor to the increased strandings that led to the declaration of the Unusual Mortality Event. Nevertheless, if MSS dolphins’ immune systems were compromised due to the DWH oil exposure [90], an increased exposure to morbillivirus and/or other marine pathogens could have led to even higher mortality.

If the MSS dolphin population is a closed population, the low dolphin survival and low reproductive success [29] would have led to a decline in the population size over the 24 months of the study. Without temporary or permanent immigration, the abundance estimates should have shown a declining trend given the number of precise density estimates (i.e. 8 estimates each with a CV < 0.10) (e.g. [91]). However, a monotonic decrease in abundance was not observed (Fig 6). The MSS abundance estimates initially ranged from 3000–3500 from July 2010 to May 2011, increased during July and October 2011 to 3800–4600 and then returned to about 3000 during January and May 2012. The peak abundances are consistent with seasonal influxes of dolphins as has been noted during other surveys [19, 41] and a lack of a warm season influx in 2010 may have been related to limited shrimp trawling. Permanent immigration by large numbers of dolphins is unlikely. Feeding specializations can vary significantly by locale and appear to primarily determine a dolphin’s habitat use and dolphins probably do not switch habitats on a routine basis (e.g. coastal to inshore or vice versa) (e.g. [92]). We conclude that our abundance results were confounded by a seasonal influx of dolphins in 2011 (but not in 2010), making it impossible to detect a decline in abundance over the relatively short study period (less than 2 years) that would be expected given the observed Unusual Mortality Event and the low estimated survival rates. Assessing the influence of seasonal movements of large numbers of dolphins on abundance patterns in MSS will be challenging. It will require the ability to prorate seasonal abundance estimates based on seasonal estimates of the proportion of year-round, seasonal and transient dolphins in MSS. These seasonal estimates will require examination of site fidelity patterns of a large number of dolphins throughout MSS.

### Movements and space use

This is the first study to use satellite-linked telemetry to assess MSS dolphin movements. Tagged dolphins had limited ranging patterns comparable to findings for many individuals from other northern GoM telemetry studies: Barataria Bay [12], St. Joseph Bay [40], Sarasota Bay, FL [93, 94], and Matagorda Bay, TX [95]. The telemetry results identified, at a minimum, two distinct communities in MSS: inshore and island (Fig 7). Additionally, none of the inshore dolphins tagged near Pascagoula Bay had 95% UDs east of Point aux Chenes while the two tagged in Grand Bay did not have 95% UDs west of Point aux Chenes. Similar fine-scale community structure has been identified in other regions of the GoM using photo-ID [67, 96] and by Wells et al. [12] in Barataria Bay using satellite-linked telemetry. While we identified an island community based on the ranging patterns of just two dolphins, both male, Wells et al. [12] identified similar community structure in Barataria Bay based on the ranging patterns of 12 female and three male dolphins (island) and 16 female and 14 male dolphins (inshore). The majority of MSS dolphins were tagged in a relatively localized area and based upon telemetry data alone, it is unlikely that they provide a comprehensive assessment of MSS community structure. Comparison of photo-ID and telemetry data provided additional insight into MSS community structure. The majority of tagged individuals was previously sighted during photo-ID surveys and had sighting locations predominantly within their respective telemetry-based 95% UDs, thus supporting the telemetry results. Although the telemetry data identified limited use of mid-MSS, there were a high number of photo-ID sightings in this region, suggesting the possibility of additional MSS communities not represented by the telemetry data. Additional satellite-linked telemetry and photo-ID studies within and outside of the study site are necessary to fully assess MSS community structure.

The hot spot analysis indicated that the Pascagoula Bay area close to shore is consistently utilized by dolphins. It also indicated that hot spots were more widespread in colder months and dolphins were slightly shifting to new areas each month (Fig 8). Tagged dolphins clustered in the Pascagoula Bay vicinity across all 5 months and exhibited a small amount of short-term seasonal shift by clustering somewhat farther south toward mid-sound during colder months and to a lesser degree, north into the Pascagoula River estuary. While this indicates a seasonal shift in distribution, the dolphins remained within the bounds of MSS. Conversely previous abundance research (e.g. [19]) has suggested a large number of dolphins completely leave MSS during colder months and move into deeper coastal waters. However, it is possible that all of the satellite tagged dolphins were year-round residents.

### Site-fidelity

The multi-year (2010–2012) photo-ID data and multi-month (~6 months) telemetry data have identified dolphins with varying levels of site fidelity to the MSS study site. A total of 162 (8.5% of total catalog) dolphins were classified as having high site fidelity and are likely year-round residents with ranges predominantly within the MSS study site (Fig 5). Based upon the telemetry data, wherein some individuals had ranges both within and beyond the photo-ID transect lines, dolphins with similar ranges are likely individuals with moderate site fidelity and resident to MSS but with ranges not confined to the boundaries of the photo-ID study area. Dolphins with low site fidelity could represent individuals that are part of the Northern Coastal Stock or adjacent MSS waters that infrequently enter the study area. Similar patterns of site fidelity have been observed in other northern GoM regions [40, 96].

MSS dolphin residence has been studied since the 1980s, and collaborations across studies have identified long-term (multi-decadal) residence of dolphins to this region as well as seasonal residents and transients [19, 24–27]. Apparent long-term site fidelity is demonstrated by the periodic resighting of some of the 57 dolphins freeze-branded in MSS during 1982–1983 [17, 18]. For example, we sighted three of these freeze-branded dolphins during our study and Hubard et al. [19] sighted two different dolphins nearly 30 and 15 years after they were branded, respectively. Long-term site fidelity was further evaluated by comparing the 2010–2012 dolphins (i.e. NRDA catalog) to dolphins with >5 sightings (n = 15) from the pre-NRDA MSS catalog begun in 1995 (1269 individuals) [19, 27]. Eight dolphins were matched in both catalogs, with initial sightings as early as 1995–2003, and had similar distributions across all sightings within the study area; five would be classified in the Island stratum and one in the Inshore stratum. Similar site fidelity patterns have been observed in other northern GoM sites with long-term residents and transients or seasonal residents that are hypothesized to be members of the adjacent Northern Coastal Stock venturing into BSE waters [40, 97]. Seasonal fluctuations in abundance have been attributed to these transients or seasonal residents entering BSE waters. Although seasonal fluctuations were not as clearly evident in this study (except for summer 2011) (Fig 6), previous research has identified a 50–100% increase in abundance in the MSS region during the warmer months compared to the cold ones [19, 23, 24]. The only winter abundance estimated during our study was generally similar to other seasons.

### Management implications

The low rate of survival estimated post-spill by the spatial robust model combined with the low rate of reproductive success determined by targeted photo-ID monitoring [29] raise significant management concerns for the Mississippi Sound, Lake Borgne, Bay Boudreau (MSLBBB) common bottlenose dolphin stock. These estimated reductions in vital rates were incorporated into a population model to compare the predicted post-spill trajectory to the trajectory for the stock that would have been expected had the DWH spill not occurred [4, 30]. Given that reproductive success was still low and health effects persisted four years post-spill when the last NRDA studies were completed [28, 29], it was uncertain how long vital rates for the exposed individuals would be affected. Therefore, expert opinion was used to estimate rates at which survival and reproduction would return to baseline. Incorporating all of this information, the population model indicated that the MSLBBB stock was one of the more highly impacted BSE stocks and that it could experience a maximum decline of 62% as compared to the expected trajectory had the DWH oil spill not occurred. Furthermore, the model predicted that in the absence of restoration, it could take 46 years for the MSLBBB stock to recover to the baseline trajectory, underscoring the critical need for effective restoration efforts.

In addition to abundance and survival estimates providing essential data to assess the impacts of the DWH oil spill, these data are also integral to understanding the susceptibility of the MSLBBB stock to other anthropogenic influences and provide insight into effective restoration planning and stock management. For management, the NMFS calculates annual potential biological removal (PBR) from an abundance estimate (N_BEST_) for each marine mammal stock. The PBR estimate is the number of non-natural mortalities (e.g. fisheries mortalities) a stock can sustain annually and remain stable or grow [98]. For stocks where abundance fluctuates seasonally, the lowest seasonal estimate is assumed to most accurately reflect the size of the year-round resident population and is typically used as the estimate for N_BEST_ because it provides the most conservative PBR. The year-round resident population is probably most susceptible to point source anthropogenic influences (e.g. commercial and recreational fishing, dredging, and environmental contaminants) due to its continuous close proximity to these point sources [13].

For the MSLBBB stock, the current N_BEST_ estimate used for management is 900 dolphins (CV = 0.63) from winter 2012 aerial surveys [13]. The lowest C-R estimate from this study for the MSS portion of the stock, 3,046 dolphins, is also from winter 2012 (January) and it would represent a large increase in PBR for the MSLBBB stock. Nevertheless, while this estimate may be positively biased to some degree for MSS proper due to lower salinity in western and eastern MSS during winter, for the MSLBBB stock it is probably conservative and currently the most accurate estimate of N_BEST_ available. The C-R study area represents 22% (782 km^2^) of the MSSLBBB stock area (3,637 km^2^) and the abundance of dolphins for just the C-R study area during January 2012 was 977 dolphins (i.e. 1.25 dolphin km^-2^ x 782 km^2^). Additionally, individual dolphins do not appear to range widely in MSS. Telemetry results indicate that the range of inshore dolphins tracked from summer to winter for an average of ~200 days is small ($\bar{x}$ < 30 km) compared to the 128 km length of MSS. It is unlikely the density estimates were influenced by different movement patterns of dolphins with respect to the C-R study area relative to the rest of MSS. The January 2012 estimate does not include dolphins inhabiting Bay Boudreau and Lake Borgne. During January 2012 NRDA dolphin biopsy sampling in Bay Boudreau, 56 dolphin groups were encountered throughout the bay ($\bar{x}$ = 6.9 dolphins/group, SE = 0.62, n = 56, range 1–22) (NMFS unpublished, [60]) and at least a small number of dolphins inhabit Lake Borgne during winter [20]. If the density of dolphins in Bay Boudreau is comparable to similar habitat in Barataria Bay, LA, as was assumed for the DWH NRDA, then over 700 dolphins inhabit Bay Boudreau [60].

The boundaries of the MSS portion of the MSLBBB stock will likely be redefined after additional research as this area may be home to multiple inshore stocks. Telemetry and photo-ID results indicate the narrow strip of coastal waters that was part of our Island stratum should be added to the current stock boundary bounded by barrier islands because island dolphins clearly used those waters. While tentative, our study indicates there are at least two dolphin communities in eastern MSS, inshore and island. Also, tagged dolphins had restricted home and core ranges that did not extend throughout MSS. This suggests fine-scale community structure within MSS similar to that described by Urian et al. [67] in Tampa Bay, FL. Whether these communities function as stocks awaits further genetic studies.

The urgent need for information on cetacean stock structure, abundance, and survival rates was evident in the recent investigation of the effects of the DWH oil spill. The findings from our study along with other DWH NRDA studies provided for the assessment of injury from the oil spill to multiple GoM cetacean stocks [4, 60]. Given the predicted impacts and size of the area affected by the spill in the northern GoM, more detailed information on stock structure is needed for prioritizing specific areas of greatest concern and greatest opportunity for restoration. Future assessments of dolphin density and abundance, and survival will be critical to evaluate the success of restoration.
